# Genetic Basis of Response of Ghanaian Local Chickens to Infection With a Lentogenic Newcastle Disease Virus

**DOI:** 10.3389/fgene.2020.00739

**Published:** 2020-07-28

**Authors:** Muhammed Walugembe, Esinam N. Amuzu-Aweh, Princess K. Botchway, Augustine Naazie, George Aning, Ying Wang, Perot Saelao, Terra Kelly, Rodrigo A. Gallardo, Huaijun Zhou, Susan J. Lamont, Boniface B. Kayang, Jack C. M. Dekkers

**Affiliations:** ^1^Department of Animal Science, Iowa State University, Ames, IA, United States; ^2^Feed the Future Innovation Lab for Genomics to Improve Poultry, Department of Animal Science, University of California, Davis, Davis, CA, United States; ^3^Department of Animal Science, University of Ghana, Accra, Ghana; ^4^Department of Animal Science, University of California, Davis, Davis, CA, United States; ^5^School of Veterinary Medicine, University of California, Davis, Davis, CA, United States

**Keywords:** NDV, GWAS, Ghanaian local ecotypes, QTL, immune response

## Abstract

Newcastle disease (ND) is a global threat to domestic poultry, especially in rural areas of Africa and Asia, where the loss of entire backyard local chicken flocks often threatens household food security and income. To investigate the genetics of Ghanaian local chicken ecotypes to Newcastle disease virus (NDV), in this study, three popular Ghanaian chicken ecotypes (regional populations) were challenged with a lentogenic NDV strain at 28 days of age. This study was conducted in parallel with a similar study that used three popular Tanzanian local chicken ecotypes and after two companion studies in the United States, using Hy-line Brown commercial laying birds. In addition to growth rate, NDV response traits were measured following infection, including anti-NDV antibody levels [pre-infection and 10 days post-infection (dpi)], and viral load (2 and 6 dpi). Genetic parameters were estimated, and two genome-wide association study analysis methods were used on data from 1,440 Ghanaian chickens that were genotyped on a chicken 600K Single Nucleotide Polymorphism (SNP) chip. Both Ghana and Tanzania NDV challenge studies revealed moderate to high (0.18 – 0.55) estimates of heritability for all traits, except viral clearance where the heritability estimate was not different from zero for the Tanzanian ecotypes. For the Ghana study, 12 quantitative trait loci (QTL) for growth and/or response to NDV from single-SNP analyses and 20 genomic regions that explained more than 1% of genetic variance using the Bayes B method were identified. Seven of these windows were also identified as having at least one significant SNP in the single SNP analyses for growth rate, anti-NDV antibody levels, and viral load at 2 and 6 dpi. An important gene for growth during stress, CHORDC1 associated with post-infection growth rate was identified as a positional candidate gene, as well as other immune related genes, including VAV2, IL12B, DUSP1, and IL17B. The QTL identified in the Ghana study did not overlap with those identified in the Tanzania study. However, both studies revealed QTL with genes vital for growth and immune response during NDV challenge. The Tanzania parallel study revealed an overlapping QTL on chromosome 24 for viral load at 6 dpi with the US NDV study in which birds were challenged with NDV under heat stress. This QTL region includes genes related to immune response, including TIRAP, ETS1, and KIRREL3. The moderate to high estimates of heritability and the identified QTL suggest that host response to NDV of local African chicken ecotypes can be improved through selective breeding to enhance increased NDV resistance and vaccine efficacy.

## Introduction

Newcastle disease (ND) is caused by a virulent pathotype of an avian paramyxovirus type 1 (APMV-1) that belongs to the family *Paramyxoviridae*, subfamily *Avulavirus*, and genus *Orthoavulavirus* (Mayo, 2002; Davison et al., 2019). It is a devastating poultry disease that causes various clinical signs in infected chickens, which vary with the pathotype of the viral strain (Marshal et al., 2009). Newcastle disease virus (NDV) strains are categorized into three pathological categories: lentogenic, mesogenic, and velogenic (Songhua et al., 2003; Brown and Bevins, 2017). Lentogenic strains are often used as vaccines and are characterized by subclinical infections or respiratory disease (Cornax et al., 2012). Mesogenic strains are characterized by low mortality, especially in young chickens, and can also cause respiratory and neurological disease. Virulent or velogenic strains often cause systemic infections, with several clinical signs, including diarrhea, neurologic disease, hemorrhagic lesions in the gastro-intestinal tract, and sudden death (Miller et al., 2010). NDV is often transmitted among flocks through fecal-oral and respiratory routes and can also be spread by other poultry species, wild animals, wild and feral birds, domestic animals, and communal water reservoirs (Awan et al., 1994).

Despite several advances made in diagnostics and vaccines for ND, the disease continues to be a major challenge to poultry producers worldwide (Alexander et al., 2012). In developing countries, particularly in Africa, local chicken production is characterized by low input production systems (Guèye, 1998). Local chickens are important for the provision of quality proteins and are a major contributor to household income, particularly for women (Alabi et al., 2006). Outbreaks of velogenic NDV strains in local chicken populations negatively impact economic livelihoods and human welfare by reducing food supplies (Alders, 2014). In Africa, local chickens are raised in free range scavenging and backyard systems (Goromela et al., 2006). As a result, African local chicken ecotypes are adapted to extreme climatic conditions with low inputs of feed and veterinary services, as evidenced by the presence of selection signatures across the genomes of local chickens (Fleming et al., 2016, 2017; Walugembe et al., 2019a). Although local chickens are well adapted to their environment and some unvaccinated local chickens have been reported to have anti-NDV antibodies (Boakye et al., 2016), velogenic ND outbreaks often lead to loss of entire chicken flocks (Guèye, 2002; Copland et al., 2018) due to lack of or ineffective vaccination. Although effective NDV vaccines are available, their use and effectiveness in multi-age backyard/scavenging chicken flocks with inadequate biosecurity and husbandry practices are very limited (Goromela et al., 2006; Alders, 2014). Moreover, there is a lack of organized breeding programs, in particular for local chicken ecotypes. As a result, local chicken ecotypes/breeds that are resilient to ND infection have not yet been developed.

Previous studies have demonstrated a genetic basis of response to lentogenic NDV infection in commercial high and low growth intercross broiler lines (Yonash et al., 2001; Luo et al., 2013) and in commercial egg-laying chickens (Rowland et al., 2018; Saelao et al., 2019). Other studies have also reported genetic parameters for some NDV response traits in local chickens (Lwelamira et al., 2009; Lwelamira, 2012). As part of a large-scale study to understand the genetic basis of NDV response traits for local chicken ecotypes in Africa (under the Feed the Future Innovation Lab for Genomics to Improve Poultry), we recently reported estimates of genetic parameters and genome-wide association study (GWAS) results for response to lentogenic NDV infection in three Tanzanian local chicken ecotypes (Walugembe et al., 2019b). Here, we report results from a parallel study that was conducted with three Ghanaian local chicken ecotypes. Prior to these lentogenic NDV infection studies in Tanzania and Ghana, similar controlled lentogenic NDV infection experiments were conducted on commercial egg-laying chickens in the US, using NDV infection only (Rowland et al., 2018) or NDV infection under heat stress (Saelao et al., 2019), with the latter more closely simulating conditions in Africa. Estimates of genetic parameters and the discovery of genomic information from this comprehensive suite of studies can provide a platform for the development of a selective breeding program to produce local chicken populations that perform favorably in harsh climatic conditions with endemic NDV.

## Materials and Methods

### Experimental Design

All experimental protocols used in this study were approved by the University of California, Davis Institutional Animal Care and Use Committee (#17853).

This study was designed similar to a parallel study conducted in Tanzania as described by Walugembe et al. (2019b). In brief, local breeder chickens were randomly sampled from three ecological zones of Ghana, namely Interior Savannah (IS), Coastal Savannah (CS), and Forest (FO) zones. Breeder birds were vaccinated for various poultry diseases, following recommendations by project veterinary personnel, with the exception of receiving no NDV vaccine. Local breeder chickens were grouped for natural mating to produce 25 sire half-sib families per ecotype, using a mating ratio of 1 sire to 8 dams to generate chicks for the NDV challenge study. Challenge experiments were conducted for a total of 1,440 chicks (411 IS, 511 CS, and 518 FO) from hatch to 38 days of age (doa) across four replicates (hatches). All chicks were raised under similar conditions with *ad libitum* access to feed and water. Chicks were challenged with a live attenuated type B1 LaSota lentogenic NDV strain at 28 days of age and evaluated for pre- and post-infection growth rate, antibody level in at 10 dpi, and viral load from tears at 2 and 6 dpi, as described by Walugembe et al. (2019b).

### Genotypes and Population Structure

Blood samples were collected using Whatman FTA cards (Sigma-Aldrich, St. Louis, MO, United States) from chicks before challenge. Genotyping and genotype quality control were as described by Walugembe et al. (2019b). A total of 403,165 SNPs with call rate > 99% and minor allele frequency > 0.05 remained after filtering (Table 1). Imputation of missing genotypes (<1% after quality control) for the 403,165 SNPs was performed using Fimpute (Sargolzaei et al., 2014). Because dams were housed in group pens, the 1,440 birds were assigned to half- and full-sib families based on their genomic relationships, with cutoffs of 0.18–0.37, 0.38–0.77, and <0.18 for half-sibs, full-sibs, and less related individuals, respectively, which were determined based on the distribution of the genomic relationships among the 1440 birds.

**TABLE 1:** Genotype quality metrics provided by Affymetrix and the requirements used in quality control filtering.

Affymetrix genotype metric	Metric description	Requirement
Nclus	Number of genotype clusters	≥2
CR	% of samples with genotype call other than “No call” for SNP	≥99%
MinorAlleleFrequency	Min (PA, PB), where PA is frequency of Allele A, and PB = 1-PA	≥0.05
FLD	Measure of the cluster quality of a probeset	≥5.13
HomRO	Distance to zero in the Contrast dimension (X position) from the center of the homozygous cluster that is closest to zero	≥0.29
HomFLD	Version of FLD computed for the homozygous genotype clusters	≥13.33
HetSO	Measures how far the heterozygous cluster center sits above the homozygous cluster centers in the Size dimension (Y position)	≥-0.21
ConversionType	Probeset classification	≠OTV
BB.varX	Contrast (X position) variance for BB cluster	≤0.85
BB.varY	Size (Y position) variance for BB cluster	≤0.68
AB.varX	Contrast (X position) variance for AB cluster	≤0.73
AB.varY	Size (Y position) variance for AB cluster	≤0.73
AA.varX	Contrast (X position) variance for AA cluster	≤0.76
AA.varY	Size (Y position) variance for AA cluster	≤0.60

The combined genotype data from the current study and that from the three Tanzanian local chicken ecotypes from the parallel study (Walugembe et al., 2019b) were used to examine the population structure within and across the two country populations using the PLINK v1.9 software (Chang et al., 2015). Shared ancestry of the Ghanaian local chicken ecotypes was explored using the Admixture software (Alexander et al., 2009), allowing the number of ancestral subpopulations to range from 1 to 6, with 3 ancestral subpopulations giving the lowest cross-validation error. The ancestral subpopulation proportions generated by admixture analyses for each individual bird were used as covariate effects in the downstream genetic analyses.

### Genetic Parameters

Variance component estimates and heritabilities were performed using ASReml 4 (Gilmour et al., 2015), as described by Walugembe et al. (2019b), with some modifications to the model fitted to account for the presence of three rather than two ancestral subpopulations. The univariate mixed linear animal model was;

(1) $$Y_{i\,j\,l\,k\,m\,n}=\mu +D_{i}+R_{j}+S_{l}+C_{k}+P_{m}+A_{n}+M_{o}+e_{i\,j\,l\,k\,m\,n\,o}$$

where Y is the dependent phenotype variable: pre- and post-infection growth rate, antibody at 10 dpi, viral load at 2 dpi, and viral load at 6 dpi. Fixed effects included death prior to 10 dpi (*D* = 0/1), replicate (*R* = 1 to 4), and sex (*S* = male/female). Two covariates, ancestral subpopulation proportions (C and P) obtained from admixture analyses were fitted. Random effects included animal genetic effects (A) with a genomic relationship matrix computed based on method 1 of VanRaden (2008), dam (M) to account for maternal effects, and residuals (e). The dam effect (based on assigned full-sib families). The dam effect was removed for some traits for which it explained no variance. For viral load at 2 and 6 dpi, and antibody at 10 dpi, qPCR plate (55 and 57 plates, for 2 and 6 dpi, respectively) and replicate plate (40), respectively, were added as fixed effects. Phenotypic variance was obtained as the sum of variance due to animal, dam, and residuals. Heritability was computed as the ratio of the estimates of animal to phenotypic variance. Bivariate animal models were used to estimate pairwise phenotypic and genetic correlations between traits, with the same fixed and random effects as specified for the univariate analyses.

### Genome-Wide Association Analyses

Two whole genome association analysis methods were used for GWAS, as described by Walugembe et al. (2019b). These two method were utilized to identify any overlap in results to better understand the sensitivity of the underlying model assumptions. Briefly, method Bayes B (Habier et al., 2011), as implemented in the Gensel software (Fernando and Garrick, 2008), was used to estimate the genetic variance accounted for each one megabase (Mb) window of SNPs. Model (1) was used and effects were fitted as described in Walugembe et al. (2019b). One Mb regions that explained more than 1% of the genetic variance explained by all SNPs across the genome were considered significant. Gene annotation for the 1-Mb windows was completed using the Genome Data Viewer in NCBI on a Gallus gallus 5 genome version^1^.

The R package GenABEL was used to identify single SNPs associated with the various NDV response traits (Aulchenko, 2015) using a hierarchical generalized linear model (Rönnegård et al., 2010). Model (1) was used for single-SNP analyses, with genotype at each SNP included as a fixed effect, one at a time. Genome-wide significance thresholds of 20 and 10% were derived based on a modified Bonferroni correction, as 0.2 or 0.1 divided by the number of independent tests (Rowland et al., 2018; Saelao et al., 2019; Walugembe et al., 2019b). A more relaxed significance threshold was used to reduce the number of false negatives and allow results to be compared to those from the Bayesian analysis and with results from previous and future studies.

## Results

### Population Structure

**FIGURE 1:**
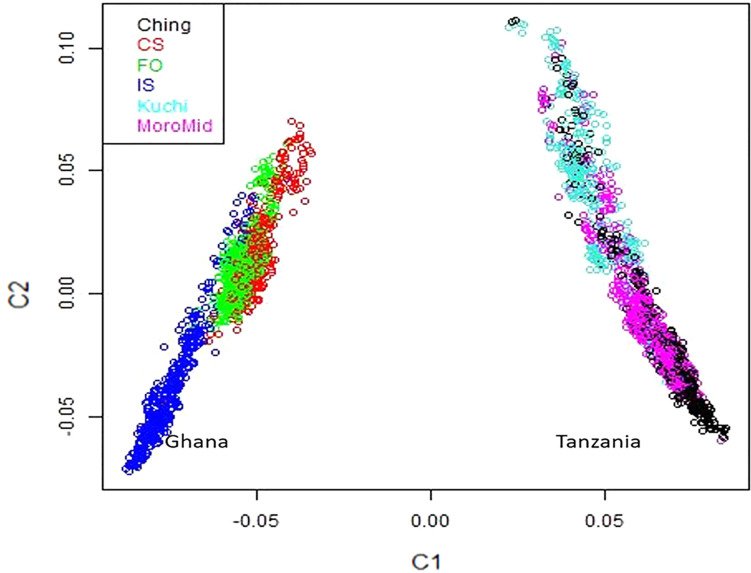
Multi-dimensional scaling plot showing distinct separation between the Ghana and Tanzania ecotypes.

**FIGURE 2:**
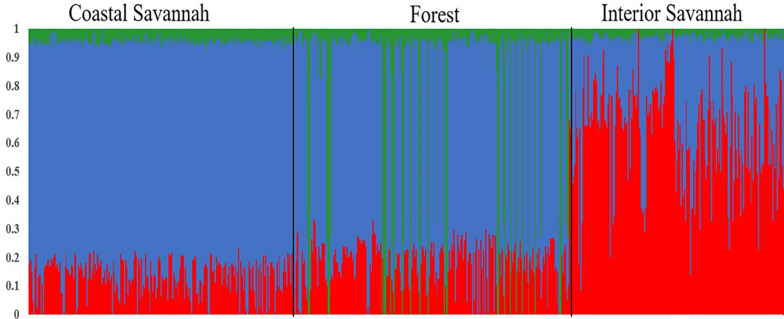
Admixture plot showing mixed ancestry among birds from the three Ghana ecotypes: Coastal Savannah, Forest, and Interior Savannah.

A multi-dimensional scaling plot of the three Ghanaian ecotypes and the three Tanzanian ecotypes evaluated in the parallel study by Walugembe et al. (2019b) (Figure 1) showed distinct clustering of the Tanzania and Ghana populations, with no overlap among ecotypes between populations from the two countries. Among the Ghanaian ecotypes, Figure 1 indicated some overlap between the three ecotypes but with FO and CS clustering separately from IS, which agreed with Admixture analyses based on identity by state (Figure 2). Although there was evidence of admixture, birds appeared to have originated from three ancestral subpopulations. Ecotypes CS and FO had a higher average proportion of subpopulation 2 (0.80 and 0.69, respectively) and a lower proportion of subpopulation 1 (0.17 and 0.20, respectively). IS had a higher proportion (0.72) of subpopulation one and the lowest proportion of subpopulation 3 (0.02).

### Genetic Parameters

Genetic parameters for the NDV response traits are presented in Table 2. Single and bivariate trait analyses revealed similar heritability estimates for the various NDV response traits. Estimates of heritabilities were moderate to high, ranging from 0.23 for viral load at 6 dpi to 0.55 for pre-infection growth rate (Table 2). There was evidence of maternal effects for growth rate post infection and for anti-NDV antibody levels at 10 dpi.

**TABLE 2:** Trait estimates (±SE) of variance components in the Ghana local chicken ecotypes population.

Trait	*N*^3^	Mean^4^	*SD*^5^	Heritability	Dam	Residual
Pre-infection GR^1^	1436	6.26	1.16	0.55 ± 0.05	0.03 ± 0.01	0.47 ± 0.04
Post-infection GR^1^	1400	7.93	2.81	0.42 ± 0.05	0.01 ± 0.01	3.77 ± 0.30
Log_10_Antibody titer^2^	1425	3.45	0.40	0.29 ± 0.05	0.02 ± 0.01	0.10 + 0.01
Log_10_Viral load, 2 dpi^2^	1416	4.31	0.85	0.41 ± 0.05	0.00 ± 0.00	0.32 ± 0.03
Log_10_Viral load, 6 dpi^2^	1408	2.94	1.02	0.23 ± 0.05	0.00 ± 0.00	0.41 ± 0.03
Viral clearance	1386	0.27	0.29	0.25 ± 0.05	0.00 ± 0.00	0.60 ± 0.00

*^1^Growth rate; ^2^Traits log_10_ transformed, dpi, day post infection; VL, viral load; ^3^number of phenotypic records; ^4^arithmetic mean; ^5^standard deviation, Viral clearance = (Log_10_Viral load, 2dpi – Log10Viral load, 6 dpi)**/**Log10Viral load, 2 dpi.*

For phenotypic correlations, pre- and post-infection growth rates were moderately positively correlated, 0.41 ± 0.03 (Table 3). Viral load at 2 dpi was positively correlated with viral load at 6 dpi (0.24 ± 0.03) and with antibody level (0.11 ± 0.03). Viral clearance was positively and highly negatively correlated with viral load at 2 and 6 dpi, with estimates of 0.34 ± 0.03 and −0.63 ± 0.02, respectively. The correlations between viral clearance with growth rate and antibody levels were low and not significantly different from zero.

**TABLE 3:** Estimates (±SE) of genetic (above diagonal) and phenotypic (below diagonal) correlations for the Ghana local chicken ecotypes population based on bivariate analyses.

	Pre-infection_GR	Post- infection_GR	Antibody	VL2 dpi	VL6 dpi	Viral Clearance
Pre-infection_GR^1^		0.53 ± 0.07	0.13 ± 0.10	0.04 ± 0.09	−0.01 ± 0.12	0.03 ± 0.11
Post_infection_GR^1^	0.41 ± 0.03		0.18 ± 0.12	−0.02 ± 0.11	0.24 ± 0.13	−0.22 ± 0.12
Antibody^2^	0.06 ± 0.03	0.04 ± 0.03		0.23 ± 0.12	0.18 ± 0.15	0.23 ± 0.15
VL2dpi^2^	0.02 ± 0.03	0.02 ± 0.03	0.11 ± 0.03		0.49 ± 0.11	0.23 ± 0.13
VL 6dpi^2^	−0.10 ± 0.03	−0.02 ± 0.03	0.07 ± 0.03	0.24 ± 0.03		−0.48 ± 0.11
Viral Clearance	0.07 ± 0.03	0.03 ± 0.03	0.04 ± 0.03	0.34 ± 0.03	−0.63 ± 0.02	

*^1^Growth rate; ^2^Traits log_10_ transformed, dpi, day post infection; VL, Viral load. Viral clearance = (Log_10_Viral load, 2 dpi – Log10Viral load, 6 dpi)**/**Log10Viral load, 2 dpi.*

The estimate of the genetic correlation between pre- and post-infection growth rate was high, 0.53 ± 0.07 (Table 3). Viral load at 2 dpi was highly genetically correlated with viral load at 6 dpi (0.49 ± 0.11). Viral clearance was positively correlated with viral load at 2 dpi (0.23 ± 0.13), but negatively correlated with viral load at 6 dpi (−0.48 ± 0.11). Antibody level at 10 dpi was genetically positively correlated with viral load at 2 dpi (0.23 ± 0.12) and at 6 dpi (0.18 ± 0.15) and with viral clearance (0.23 ± 0.15); birds with greater antibody levels post challenge had greater viral clearance. Antibody levels were positively correlated with pre- (0.13 ± 0.10) and post- (0.18 ± 0.12) infection growth rates. Post-infection growth rate was positively correlated with viral load at 6 dpi (0.24 ± 0.13) but not significantly different from zero for viral load at 2 dpi. However, genetic correlation estimates had substantial standard errors.

### Association Analyses

After quality control, a total of 1,440 animals and 403,165 SNPs remained and were used for GWAS analyses. Due to the clear evidence of admixture, analyses were performed across all three Ghana ecotypes by fitting ancestral subpopulation proportions (C and P) to account for population structure. A total of 72,317 principal components (pc) accounted for 95% of the variance between SNPs genotypes, resulting in 10 (49,271 pc) and 20% (28,834 pc) genome-wide p-value thresholds of 2.02 × 10^–6^ and 6.94 × 10^–6^, respectively, to declare suggestive associations for the single SNP GenABEL analyses.

Results of the Bayes B and single-SNP GWAS are shown in Tables 4, 5, respectively. One-Mb windows that explained more than 1% of genetic variance in Bayes B corresponded in location to 7 QTL identified in the single-SNP analysis across all traits analyzed. In the following, results will be described by trait by first reporting Bayes B results, followed by a description of any corresponding single-SNP analysis results in terms of location.

**TABLE 4:** Percentage of genetic variance explained by 1-Mb genomic regions that are associated with NDV response traits in the Ghana local chicken ecotypes population based on the Bayes-B method.

Trait	Chr	Position window (Mb)	# markers	%GV^1^
Pre-infection GR	15	8001139 – 8999298	645	2.1
	1	169001420 – 169998993	350	1.4
	2	47000713 – 47999595	335	1.1
	2	31002183 – 31998250	301	1.0
	21	4000320 – 4997196	905	0.9
	14	49515 – 999797	513	0.8
	27	2000333 – 2998886	1143	0.8
	5	15000770 – 15998814	404	0.7
	21	5001208 – 5998828	1138	0.6
	11	19001310 – 19997265	394	0.51
Post-infection GR	1	186000674 – 186992961	406	13.11
	2	33002170 – 33998193	306	1.35
	4	76002754 – 76999095	291	1.29
	6	29001552 – 29999994	471	0.56
	1	120003806 – 120999766	362	0.54
	2	126002189 – 126997805	343	0.50
Antibody^2^	17	7000143 – 7999150	686	1.46
	4	88001916 – 88997646	361	1.0
	1	165000717 – 165999016	407	0.81
	21	5001208 – 5998828	1138	0.71
	7	15001757 – 15998731	392	0.68
	13	9001252 – 9999946	519	0.58
	13	13000987 – 13998680	455	0.58
	2	33002170 – 33998193	306	0.50
Log_10_Viral load, 2 dpi^2^	13	8000885 – 8998322	409	3.16
	1	192000815 – 92994499	399	2.53
	2	104002020 – 104998000	312	2.19
	1	152000904 – 152996463	340	2.16
	27	4081455 – 4999960	480	1.86
	13	9001252 – 9999946	519	1.37
	15	9002743 – 9999015	632	1.03
	10	11000334 – 11998191	722	0.8
	8	23002812 – 23996478	477	0.76
	13	7003772 – 7998970	518	0.74
	11	5000641 – 5998062	501	0.56
	1	131002677 – 131998418	388	0.51
Log_10_Viral load, 6 dpi^2^	7	8003124 – 8997158	283	2.03
	13	9001252 – 9999946	519	1.37
	2	41001878 – 41999433	308	1.21
	5	42000579 – 42998677	381	1.12
	7	7006436 – 7999380	115	1.11
Log10Viral load, 6 dpi^2^	10	4000118 – 4996985	700	0.97
	4	22003900 – 22989145	329	0.84
	9	17001007 – 17999967	609	0.67
	10	3000312 – 3999247	677	0.62
	12	16002769 – 16999550	504	0.57
	2	26004727 – 26999450	297	0.54
	19	8216 – 996465	663	0.51
	5	32001226 – 32997980	261	0.50
Viral Clearance	9	22000680 – 22998001	579	0.82
	2	26004727 – 26999450	297	0.68
	19	8216 – 996465	663	0.63
	2	25002058 – 25998884	287	0.6
	1	178002574 – 178999573	408	0.6
	21	3002374 – 3998996	939	0.5
	3	109001864 – 109997943	413	0.5

*^1^Percentage of genetic variance; ^2^Traits log_10_ transformed.*

**TABLE 5:** Single nucleotide polymorphisms (SNPs) associated with NDV response traits in the Ghana local chicken ecotypes population, along with positional candidate genes and previously reported QTL.

Trait	SNP	Position	*P*-value	Candidate genes and location
Pre-infection_GR	AX-75846279 AX-77227276 AX-76359725	15: 8366548 Z: 47492145 27:2997424	6.76E-07 4.65E-06 6.53E-06	SNRPD3, downstream, 109047 TBX6, downstream, 70379 PJA2, downstream, 92654 FER, downstream, 16472 SLC25A46, upstream, 654900 EFNA5, upstream, 555437 DDX42, intron
Post-infection_GR	AX-75376474 AX-76076573	1:186816361 2: 33177747	3.03E-06 9.50E-06	FAT3, upstream, 33316 CHORDC1, upstream, 819564 JAZF1, intron
Antibody levels	AX-75874883 AX-76984929 AX-76057582	17: 7540201 7: 15210255 2: 22833814	4.98E-06 6.04E-06 8.64E-06	VAV2, intron CCDC141, downstream, 14125 CWC22, downstream, 349033 MIR7474, upstream, 202614 FAM133B, intron CDK6, intron
Log_10_Viral load, 2 dpi	AX-75297835 AX-75297834 AX-76244242	1:152239826 1:152239806 21:337234	1.70E-06 8.54E-06 5.99E-06	SPRY2, upstream, 430199 MIR17, upstream, 4215748 SPRY2, upstream, 430179 MIR17, upstream, 4215768 VAMP3, upstream, 41376 PHF13, downstream, 203869
Log_10_Viral load, 6 dpi	AX-76842268 AX-80796104 AX-77054277	5:42915865 9:17913643 7:9460193	3.02E-06 4.85E-06 7.36E-06	GALC, intron ZC3H14, upstream, 250637 GTF2A1, upstream, 2021161 PIK3CA, upstream, 299514 TBL1XR1, upstream, 77713 STK17B, downstream, 339147 STAT4, upstream, 1477700
Viral Clearance	AX-76103078 AX-76755933 AX-77162202	2:48584337 4: 8693172 9: 22887909	3.60E-006 6.63E-06 1.28E-05	ITGA9, downstream, 291683 POF1B, upstream, 3414 APOOL, downstream, 65714 MIR6704, downstream, 12770 LEKR1, downstream, 7369 PTX3, upstream, 65617 SSR3, upstream, 94802

**FIGURE 3:**
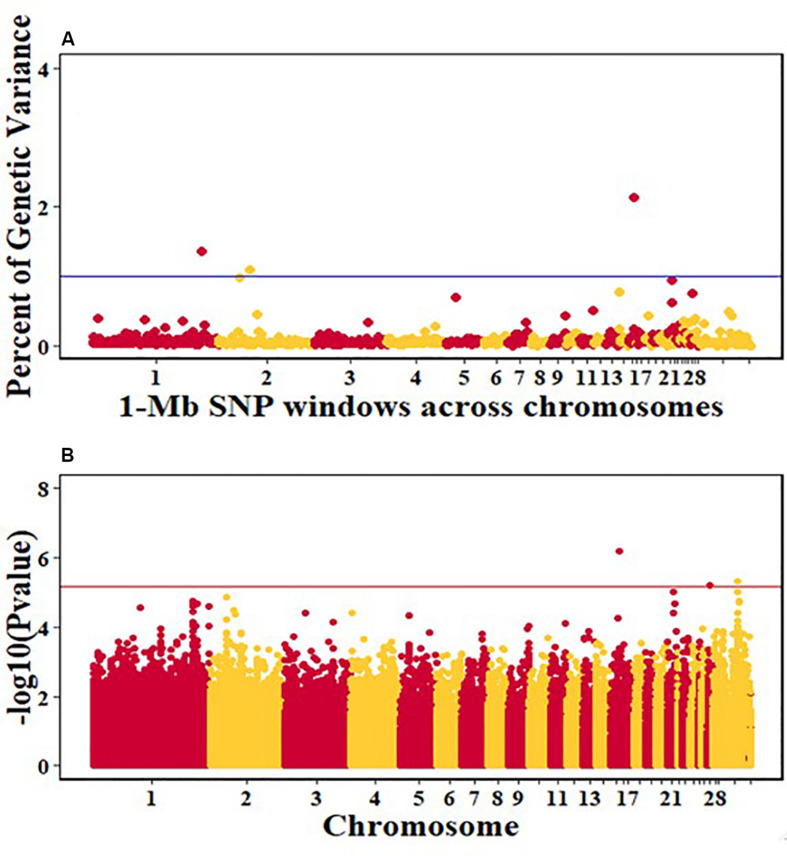
Manhattan plots showing the genome-wide association results for pre-infection growth rate in the Ghana local chicken ecotypes population, using Bayes-B and single-SNP analyses. **(A)** Bayes-B results showing the percent of genetic variance explained by SNPs in 1-Mb non-overlapping window across chromosomes. **(B)** Single-SNP results showing the -log10(*p*-value) of ordered SNPs across the chromosomes. The blue and red lines indicate genome-wide significance at 1% genetic variance and 20% genome-wide suggestive association, respectively.

**FIGURE 4:**
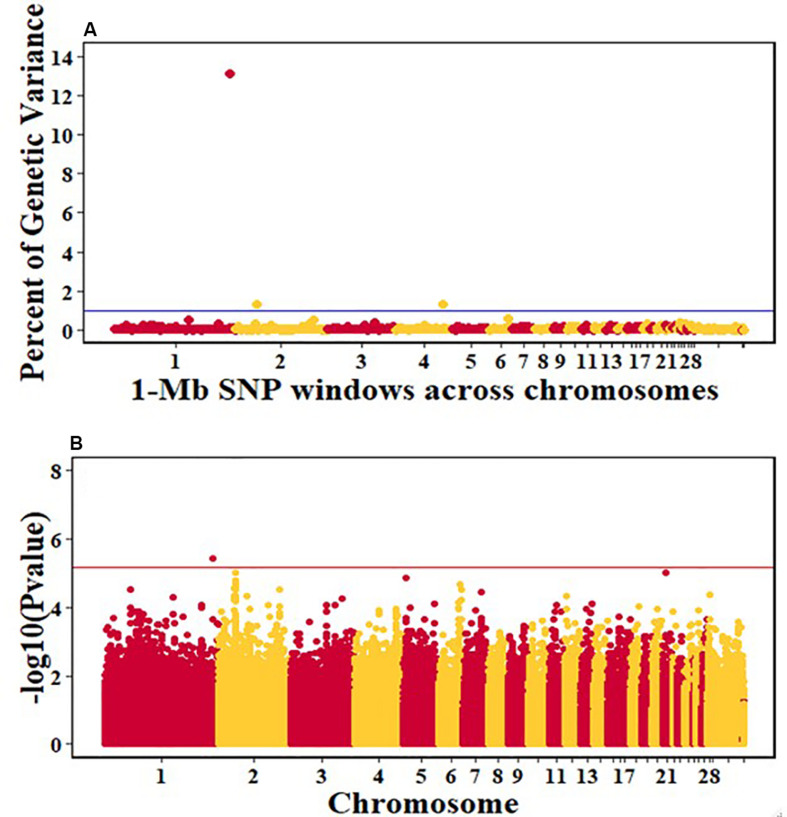
Manhattan plots showing the genome-wide association results for post-infection growth rate in the Ghana local chicken ecotypes population, using Bayes-B and single-SNP analyses. **(A)** Bayes-B results showing the percent of genetic variance explained by SNPs in 1-Mb non-overlapping windows across chromosomes. **(B)** Single-SNP results showing the -log10(Pvalue) of ordered SNPs across the chromosomes. The blue and reds lines indicate genome-wide significance at 1% genetic variance and 20% genome-wide suggestive association, respectively.

**FIGURE 5:**
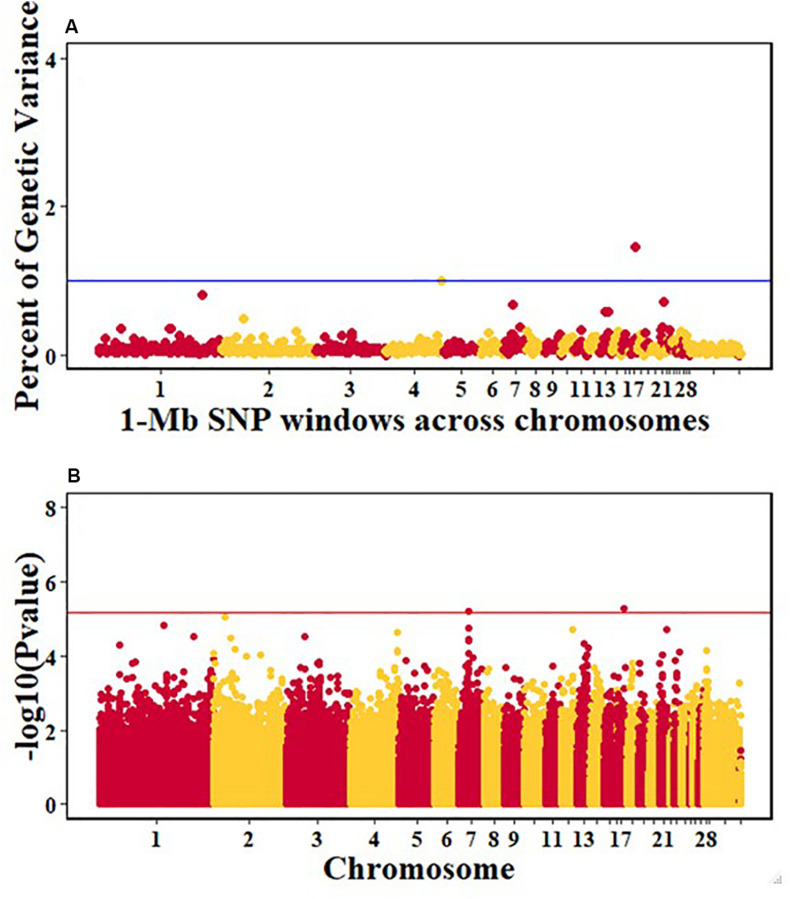
Manhattan plots showing the genome-wide association results for antibody levels at 10 dpi in the Ghana local chicken ecotypes population, using Bayes-B and single-SNP analyses. **(A)** Bayes-B results showing the percent of genetic variance explained by SNPs in 1-Mb non-overlapping windows across chromosomes. **(B)** Single-SNP results showing the -log10(Pvalue) of ordered SNPs across the chromosomes. The blue and red lines indicate genome-wide significance at 1% genetic variance and 20% genome-wide suggestive association, respectively.

**FIGURE 6:**
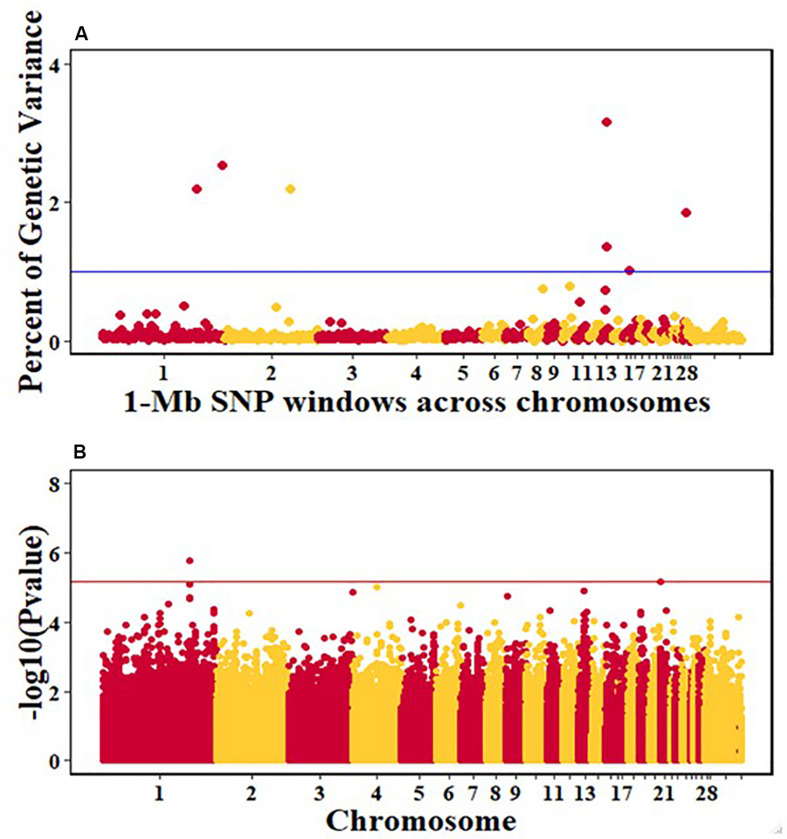
Manhattan plots showing the genome-wide association results for viral load at 2 dpi in the Ghana local chicken ecotypes population, using Bayes-B and single-SNP analyses. **(A)** Bayes-B results show the percent of genetic variance explained by 1-Mb non-overlapping windows of SNPs across chromosomes. **(B)** Single-SNP results show the -log10(Pvalue) of ordered SNPs across the chromosomes. The blue and red lines indicate genome-wide significance at 1% genetic variance and 20% genome-wide suggestive association, respectively.

**FIGURE 7:**
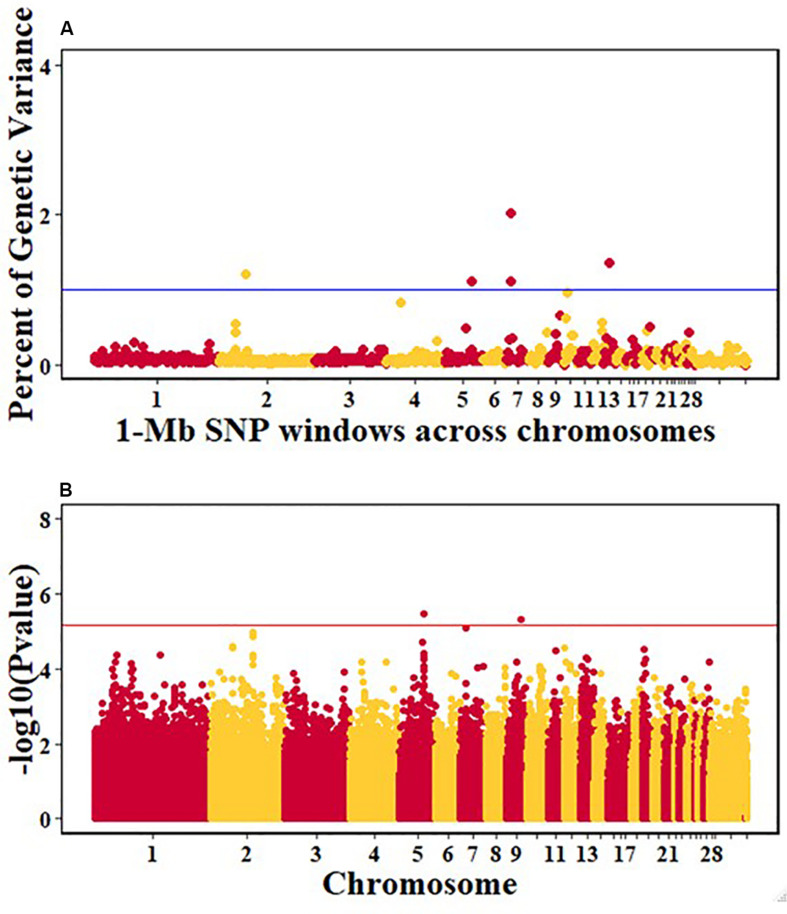
Manhattan plots showing the genome-wide association results for viral load at 6 dpi in the Ghana local chicken ecotypes population, using Bayes-B and single-SNP analyses. **(A)** Bayes-B results showing the percent of genetic variance explained by SNPs in 1-Mb non-overlapping windows across chromosomes. **(B)** Single-SNP results showing the -log10(Pvalue) of ordered SNPs across the chromosomes. The blue and red lines indicate genome-wide significance at 1% genetic variance and 20% genome-wide suggestive association, respectively.

**FIGURE 8:**
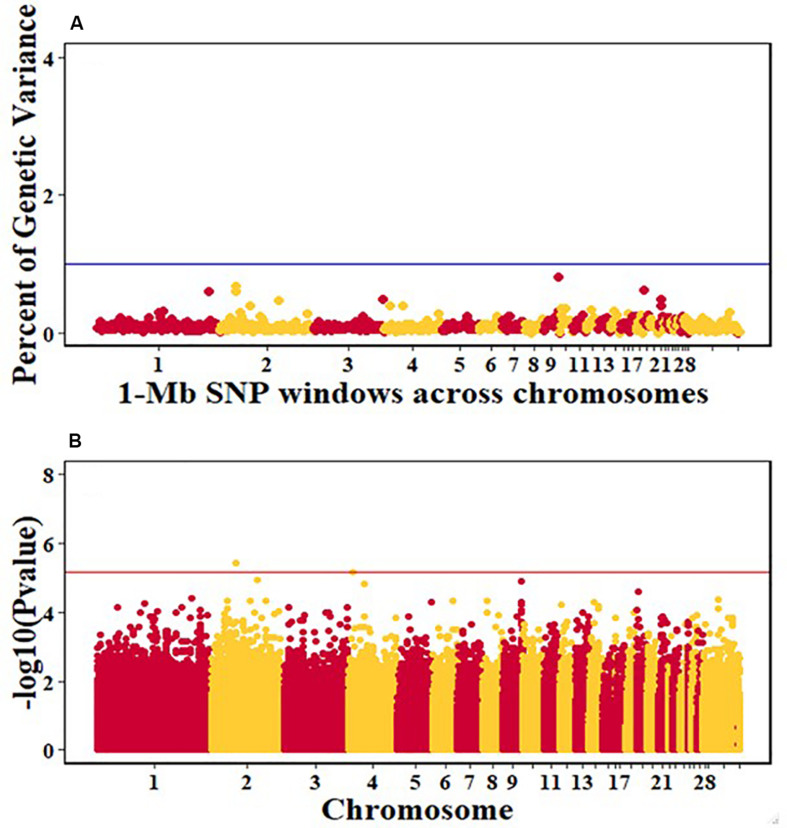
Manhattan plots showing the genome-wide association results for viral clearance in the Ghana local chicken ecotypes population, using Bayes-B and single-SNP analyses. **(A)** Bayes-B results showing the percent of genetic variance explained by SNPs in 1-Mb non-overlapping windows across chromosomes. **(B)** Single-SNP results showing the -log10(Pvalue) of ordered SNPs across the chromosomes. The blue and red lines indicate genome-wide significance at 1% genetic variance and 20% genome-wide suggestive association, respectively.

For pre-infection growth rate, 10 1-Mb windows on 8 chromosomes each captured more than 0.5% of the genetic variance, with a total of 9.9% of genetic variance explained. The window with the highest genetic variance (2.1%) was co-located with a suggestive SNP from the single SNP analyses on chromosome 15 (Figure 3). For post-infection growth rate, 6 associated windows on 4 chromosomes jointly explained 17.4% of genetic variance. One suggestive SNP from the single SNP analyses on chromosome 1 co-located with a 1-Mb window that explained 13.1% of the genetic variance (Figure 4). Eight associated windows on 7 chromosomes jointly explained 6.3% of the genetic variance for anti-NDV antibody level at 10 dpi. One significant SNP located in the intron of the VAV gene (Table 5) corresponded in location with a window that explained the most genetic variance (1.5%) for antibody level (Figure 5). For viral load at 2 dpi, 12 associated windows on 9 chromosomes jointly explained 17.7% of the genetic variance. Two significant SNPs on chromosome 1 (Figure 6) both corresponded to one 1-Mb window that explained 2.2% of genetic variance for viral load at 2 dpi. There were 13 associated windows on 10 chromosomes that jointly explained 12.1% of the genetic variance for viral load at 6 dpi (Figure 7). One suggestive significant SNP corresponded in location with a 1-Mb window that explained 1.1% of genetic variance for viral load at 6 dpi. A 1-Mb window that explained 1.4% of the genetic variance on chromosome 13 was detected for both viral load at 2 and 6 dpi. For viral clearance, 7 associated windows were identified on 6 chromosomes, which jointly explained 4.3% of the genetic variance, as well as two suggestive significant SNPs based on single SNP analyses, on chromosomes 2 and 4. These SNPs did not correspond in location with windows identified by Bayes B (Figure 8).

## Discussion

### Population Structure

The three Ghana local ecotypes evaluated here showed clear genetic separation from the three Tanzania populations evaluated in the parallel study reported by Walugembe et al. (2019b), indicating limited benefits from joint analyses of the results from these two studies. Evidence of admixture between the Ghana ecotypes indicated overlapping genetic backgrounds of the three Ghana local chicken ecotypes CS, IS, and FO (Figure 2), similar to what was found for the three Tanzania ecotypes investigated in Walugembe et al. (2019b). These admixtures are likely the result of movement of birds between regions and some inter-breeding of chickens from different regions. The CS and FO ecotypes were sampled from regions that were geographically neighboring each other, compared to IS, which was more distant, which could explain why the two former ecotypes had greater overlap of genetic backgrounds through subpopulation 2, as identified in the admixture analyses. The Forest and Savannah ecotypes have previously been reported as admixed based upon microsatellite marker analyses (Osei-Amponsah et al., 2010).

### Genetic Parameters

Heritability estimates were moderate to high for all traits evaluated in the Ghana populations, ranging from 0.23 for viral load at 6 dpi to 0.55 for pre-infection growth rate. Except for viral clearance, heritability estimates for NDV response traits for the Ghana populations were in agreement with those of the parallel study of Tanzania ecotypes (Walugembe et al., 2019b) and previous studies of lentogenic NDV infection in commercial egg laying chickens in the US (Rowland et al., 2018; Saelao et al., 2019). The current study is the first to report a moderate heritability estimate for viral clearance. Estimates for anti-NDV antibody levels at 10 dpi were similar to estimates in the previous and parallel studies in commercial egg laying chickens (Rowland et al., 2018) and Tanzania local chickens (Lwelamira et al., 2009; Walugembe et al., 2019b). These consistently moderate to high estimates of heri across populations and studies demonstrate that response to lentogenic NDV infection can be influenced through selective breeding to enhance resilience of chickens to NDV infection and/or improve vaccine response.

The positive genetic correlations between growth rates and anti-NDV antibody levels at 10 dpi found for Ghana ecotypes agree with results from the Tanzania local chicken ecotypes (Walugembe et al., 2019b) but not with estimates from lentogenic NDV infection of commercial laying chickens, which demonstrated very low negative genetic correlation estimates (Rowland et al., 2018). The positive correlations revealed for the Ghana and Tanzania ecotypes suggest that selection for increased antibody response in these local ecotypes would also increase post-infection growth rate. Antibody levels at 10 dpi were moderately positively correlated with viral load at 2 dpi in the Ghana ecotypes, which is in disagreement with Rowland et al. (2018), but in agreement with results by Saelao et al. (2019) for commercial layer chickens challenged with both NDV and heat stress. For the Tanzania ecotypes (Walugembe et al., 2019b), a low positive genetic correlation was found. Although a positive genetic correlation was revealed between antibody levels and viral load at 6 dpi for the current study, other studies reported negative correlations (Rowland et al., 2018; Saelao et al., 2019; Walugembe et al., 2019b). Moreover, there was positive genetic correlation between antibody levels and viral clearance. The higher antibody levels in the current study possibly result in increased viral clearance by 6 dpi. Based on our findings, we speculate that the increase in antibody levels post NDV infection is crucial in clearing the virus in African ecotypes. This is particularly important in African countries such as Ghana, where NDV is endemic and most of the local free-range birds will be infected with the virus at some time of their lives. Local chicken ecotypes are often raised under inadequate biosecurity and husbandry practices and are prone to NDV infections (Goromela et al., 2006).

### Association Analyses

For GWAS, two methods of analyses were used. The Bayes B method in the Gensel software was used to estimate the genetic variance captured by each 1-Mb window for all the NDV response traits. For the second method, the GenABEL R package was used to identify single SNPs associated with NDV response traits by fitting single SNPs one-by-one during the association analyses.

Multiple QTL were identified for the Ghana ecotypes. None of these QTL, however, overlapped with the parallel studies conducted in Tanzanian local chicken ecotypes (Walugembe et al., 2019b) and in commercial laying chickens in the US (Rowland et al., 2018; Saelao et al., 2019). However, there was overlap in the biological function of positional candidate genes identified in the different studies, as described in the following.

For post-infection growth rate, the single-SNP analyses identified one suggestive SNP for the Ghana ecotypes, for post-infection growth rate on chromosome 1 at 186.8 Mb. This QTL corresponded in location with a 1-Mb window (Table 5) that explained 13.1% of genetic variance based on the Bayes-B analyses. This region includes the CHORDC1 gene as a positional candidate, 819,564 bp upstream of the most significant SNP. The CHORD containing protein 1 (CHORDC1) is important for proper cell divisions and its overexpression in NIH3T3 cells leads to increased stress resistance of the cells (Michowski et al., 2010). CHORDC1 possesses chaperon activity and protects cells from cell death induced by different stress stimuli (Michowski et al., 2010). In plants, the CHORDC1 protein contains two evolutionary domains, Cys and His Rich, which were first identified in Rar-1, a plant protein involved in pathogen resistance signaling (Shirasu et al., 1999). The CHORDC1 gene could be important in maintaining growth rate during the NDV infection period. Another near significant QTL for post-infection growth rate was identified by the single-SNP analyses on chromosome 2. This QTL corresponded in location to a window that explained 1.4% of genetic variance. One significant SNP for this QTL was located in the intron of the JAZF1 gene. The JAZF1 gene reduces lipid synthesis and increases lipolysis in adipocytes and liver cells (Li et al., 2011). This is essential because there is need for energy for normal body function and for the immune system during infection. Although there was no overlap in QTL location between the current study and the Tanzania study for post-infection growth rate (Walugembe et al., 2019b), important genes that could be essential for the growth of birds post challenge were identified in the Tanzania study. One of the notable genes is the SBDS gene, which might be crucial in regulating cellular processes during disease challenge (Austin et al., 2005; Leung et al., 2011). The prior study of commercial birds did not identify any QTL associated with post-infection growth rate (Rowland et al., 2018).

One suggestive SNP for anti-NDV antibody levels was identified on chromosome 17 by the single-SNP analyses in the Ghana population. This SNP corresponded in location to a 1-Mb window that explained 1.5% of genetic variance based on the Bayes-B analysis. This SNP is within the intron of the VAV2 gene (Tables 5, 6), whose protein is important in regulating some of the early events in receptor signaling. VAV2 belongs to a family of proteins (1–3) that play a role in regulating lymphocyte development and function (Tartare-Deckert et al., 2001; Turner and Billadeau, 2002). The Tanzania study identified the HES1 gene as a positional candidate gene in the only genomic region that explained > 1% genetic variance for anti-NDV antibody level, on chromosome 9 (Walugembe et al., 2019b). Like VAV2, HES1 is also important in lymphocyte development (Wendorff et al., 2010). One QTL for anti-NDV antibody level in a commercial layer line was identified by Rowland et al. (2018), on chromosome 10, with three nearby positional candidate genes that are important for immune response.

**TABLE 6:** Genes located in 1-Mb windows with ≥1% of genetic variance for NDV response traits in the Ghana local chicken ecotypes population.

Trait	Chr: Window(Mb)	#SNPs	Genes
Pre-infection GR	15:8.0 – 9.0	645	IGLL1, GSTT1, SMARCB1, ADORA2A, MIF, TBX6, XBP1, DGCR2, SMPD4, UPB1, DERL3, CRKL, SNRPD3, DDT, SPECC1L, SLC2A11L3, MMP11, GSC2, GNAZ, GSTT1L, LRRC75B, SLC2A11L4, SLC2A11L2, SLC2A11, RSPH14, RAB36, BCR, GUCD1, GGT1, GGT5, KLHL22, CABIN1, DDX51, SLC2A11L1, CHCHD10, VPREB3, CA15L, SBSPONL, SUSD2, KREMEN1, ZNRF3, MED15, VPS29L, LOC416924
	1:169.0 – 170.0	350	TPT1, ESD, KCTD4, SUCLA2, LCP1, SPERT, LRRC63, HTR2A, NUDT15, RUBCNL, CPB2, SIAH3, COG3, SLC25A30, GTF2F2, LRCH1, ZC3H13, ERICH6B,
	2:47.0 – 48.0	335	TBX20, BMPER, FKBP9, NT5C3A, NPSR1, FANCD2OS, KBTBD2, BBS9, DPY19L1, RP9, TRNAM-CAU
Post-infection GR	1:186.0 – 187.0	406	MRE11, CWC15, FUT4, MTMR2, C11orf97, GPR83, AMOTL1, ENDOD1, SESN3, MAML2, CCDC82, ANKRD49, FAM76B, CEP57, PANX1, MIR6570,
	2:33.0 – 34.0	306	CHN2, HACL1, JAZF1, COLQ, TRIL, CREB5, PLEKHA8, FKBP14, SCRN1, PRR15, WIPF3
	4:76.0 – 77.0	291	FGFBP2, PROM1, LDB2, FGFBP1, TAPT1, CPEB2, C1QTNF7, CC2D2A, FBXL5, BST1, CD38, MIR1602
Antibody	17:7.0 – 8.0	686	COL5A1, OLFM1, SURF1, WDR5, GFI1B, RPL7A, MED22, RXRA, VAV2, SURF2, CEL, GBGT1, SPACA9, SURF6, FAM163B, TMEM8C, CCDC180, AK8, RALGDS, BRD3, SLC2A6, TSC1, STKLD1, REXO4, ADAMTS13, CACFD1, ADAMTSL2, SARDH, DBH, SURF4, GTF3C5, BRD3OS,
	4:88.0 – 89.0	361	PTPRA, MAVS, MRPS26, LZTS3, AVP, RNF24, OXT, SLC4A11, HTR7L, DDRGK1, UBOX5, FASTKD5, PANK2, SMOX, ADRA1D, MIR103A2, MIR146C-1
Log10Viral load, 2 dpi	13:8.0 – 9.0	409	FABP6, IL12B, GABRB2, ADRA1B, GABRA6, PTTG2, SLU7, FBXO38, ATP10B, C1QTNF2, CCNJL, PWWP2A, TTC1, HTR4, MIR458A, MIR146A, MIR458B
	1:192.0 – 193.0	399	TAS2R4
	2:104.0 – 105.0	312	CDH2, AQP4, SS18, CHST9, TAF4B, KCTD1
	1:152.0 – 153.0	340	SLITRK1
	27:4.0 – 5.0	480	ITGB3, CRHR1, FTSJ3, SMARCD2, METTL2A, A2ML4, PSMC5, TANC2, MRC2, TLK2, EFCAB3, ASIC2, PRR29, LOC771308, RPRML, TRNAS-GCU
	13:9.0 – 10.0	519	NKX2-5, STK10, CSNK1A1L, STC2, DUSP1, ADRB2, RPL26L1, RPL26, CREBRF, GRPEL2, NEURL1B, SH3PXD2B, AFAP1L1, IL17B, SH3TC2, BNIP1, ERGIC1, UBTD2, ABLIM3, PCYOX1L, HTR4, ARHGEF37, PPARGC1B, MIR3523, MIR6652, LOC416147
	15:9.0 – 10.0	632	PXN, HNF1A, LIMK2, RPLP0, MSI1, SLC5A1, PRKAB1, CABP1, YWHAH, DYL1, DRG1, SIRT4, COQ5, RNF185, RAB35, ACADS, TMEM233, DYNLL1, BICDL1, PLA2G1BL, GNAZ, SFI1, CCDC60, CIT, GCN1, PLA2G1B, COX6A1, TRIAP1, GATC, RNF10, POP5, MLEC, UNC119B, SPPL3, PIK3IP1, PATZ1, EIF4ENIF1, PISD, DEPDC5, RSPH14, MIR6673, DYL2, C15H12orf43, PRR14L
Log10Viral load, 6 dpi	7:8.0 – 9.0	283	TSSK6L2, TMEFF2, CAVIN2, NABP1, MYO1B,
	13:9.0 – 10.0	519	NKX2-5, STK10, CSNK1A1L, STC2, DUSP1, ADRB2, RPL26L1, RPL26, CREBRF, GRPEL2, NEURL1B, SH3PXD2B, AFAP1L1, IL17B, SH3TC2, BNIP1, ERGIC1, UBTD2, ABLIM3, PCYOX1L, HTR4, ARHGEF37, PPARGC1B, MIR3523, MIR6652, LOC416147
	2:41.0 – 42.0	308	NOD1, SH3BP5, RPL14, PIK3R4, MRPL3, NEK11, EAF1, ATP2C1,
	5:42.0 – 43.0	381	FLRT2
	7:7.0 – 8.0	115	PCNT, ITGB2, STAT1, ADARB1, GLS2, DIP2A, STAT4, MYO1B, FAM207A, KMO, C7H21orf58
Viral Clearance	9:22.0 – 23.0	579	RARRES1, PTX3, SMC4, MLF1, MIR15B, LXN, TRIM59, GFM1, MFSD1, KPNA4, ARL14, VEPH1, SHOX2, PPM1L, OTOL1, LEKR1, CCNL1, PQLC2L, SCHIP1, NLRP1L, IFT80, NMD3, SPTSSB, IL12A, MIR16-2, MIR1692, C9H3orf80, SPTSSBL, RSRC1, B3GALNT1, LOC112533096

Single SNP analyses revealed a significant suggestive SNP on chromosome 1 associated with viral load at 2 dpi in the Ghana population. Seven windows, each explaining > 1% of the genetic variance, were identified using Bayes-B (Table 4). However, none of these windows corresponded in location with the significant SNP from the single SNP analyses. The gene SPRY2 that was identified as a positional candidate gene in the single-SNP analyses, functions as a negative regulator of the fibroblast growth factor signaling pathway (Atomura et al., 2016). A 1-Mb window on chromosome 13 that explained 3.2% of genetic variance for viral load at 2 dpi contains the IL12B gene as a potential candidate gene. Interleukin-12 is a proinflammatory cytokine that belongs to the p40 subunit of both IL-12 and IL-23 and is secreted by dendritic and phagocytes cells. It produces interferon-gamma by T lymphocytes and natural-killer cells (Zhou et al., 2007). The Tanzania study identified a significant QTL on chromosome 5 for viral load at 2 dpi and a corresponding 1-Mb window that explained 2% of genetic variance (Walugembe et al., 2019b). This window contained multiple immune response genes, including ZFP36L1, SLC39A9, and ACTN1. For commercial layers, suggestive QTL associated with viral load at 2 dpi were identified by Saelao et al. (2019), but no QTL was identified by Rowland et al. (2018). The identified QTL regions reported by Saelao et al. (2019) contained genes such as TIRAP, ETS1, and KIRREL3 that are related to immune response. The presence of immune response genes in the identified QTL regions for viral load at 2 dpi, an early time post-infection, is an indication that the birds are responding to the NDV challenge and could signal attempts by the birds to clear the virus.

A window at 9.0 Mb on chromosome 13 that explained 1.4% of genetic variance was common for viral load at both 2 and 6 dpi in the Ghana population (Table 4). Several immune response genes are located in the region, including STK10, DUSP1, CREBRF, NUEURL, ZFP36L, and IL17B (Table 6). The DUSP1 (Dual-specific phosphatase 1) gene is a critical regulator of innate immune responses and negatively regulates two kinases, MAPK signaling pathways and p38 MAPK (Abraham and Clark, 2006; Hoppstädter and Ammit, 2019; Lang and Raffi, 2019). The DUSP genes are differentially expressed in immune cells and are a set of molecular control devices that specify and modulate MAPK signaling, leading to innate and adaptive immune effector functions (Lang et al., 2006). The parallel study of Tanzania ecotypes also indicated that viral load at 6 dpi was associated with regions that contain genes that are involved in modulating early innate immune response (Walugembe et al., 2019b). Although DUSP1 is located in a different genomic region, it serves similar functions and could be an important gene in the early or later stages of NDV infection to ensure resilience of birds against NDV. There was a common QTL on chromosome 24 for viral load at 6 dpi between the Tanzania study (Walugembe et al., 2019b) and the commercial layer study in which birds were challenged with NDV under heat stress (Saelao et al., 2019). However, there was no overlap of QTL identified in these two studies and the Ghana GWAS study. There was also no overlap between the Ghana study and the study by Rowland et al. (2018), in which the commercial layers were challenged with only NDV. This may be attributed to the genetic differences between Tanzanian and Ghanaian local chicken ecotypes (Figure 1). Additionally, differences in climate between the locations where the Tanzania and Ghana experiments were conducted may explain why there was overlap between the Tanzania and Saelao et al. (2019) studies, but not with the Ghana study for viral load at 6 dpi. Morogoro, where the Tanzania experiment was conducted, has a tropical savannah climate, while Accra, where the Ghana experimental station is located, has a hot semi-arid climate^2^. Although the yearly temperatures are nearly the same with ranges of 23–33°C and 22–32°C for Accra and Morogoro respectively, there is a difference in humidity between the two locations. Tanzania’s humidity could be closer to US Davis test conditions where Saelao et al. (2019) study was conducted compared to the Ghana.

## Conclusion

Results indicated moderate to high estimates of heritability for all NDV response traits in studies involving Africa ecotypes and US commercial laying birds. Growth rate post infection was genetically correlated with anti-NDV antibody level, while viral load at 6 dpi was positively genetically correlated with viral clearance. Twelve suggestive QTL and 20 1-Mb regions that explained > 1% of genetic variance were identified in the Ghana population. Seven QTL, containing one SNP from the single SNP analyses corresponded in location with a 1-Mb window explaining > 1% genetic variance for growth rate, anti-NDV antibody levels, and viral load at 2 and 6 dpi. Genes in these regions, including CHORDC1 and DUSP1 are important candidate genes for post-infection rate and viral load at 2 and 6 dpi, respectively, and could be crucial in the chickens’ response to NDV infection. The moderate to high estimates of heritability and the identified QTL for the NDV response traits in the Africa local chicken ecotypes indicate that NDV response can be improved through selective breeding of Africa local chicken ecotypes to enhance NDV resistance and vaccine efficacy. The QTL and identified genomic regions provide a platform for future investigations to understand the underlying molecular mechanisms of Africa local chickens’ response to NDV.

## Data Availability Statement

The datasets generated for this study can be found in EBI (Project: PRJEB39468; Analysis: ERZ1467227). The download will be made via the EBI study browser at https://www.ebi.ac.uk/eva.

## Ethics Statement

The animal study was reviewed and approved by University of California, Davis Institutional Animal Care and Use Committee.

## Author Contributions

MW analyzed the data and wrote the manuscript. PB collected the samples, isolated the DNA and viral RNA, and performed the qPCR. EA-A contributed to the data analyses. BK designed the experiment, oversaw the sample collection and laboratory assays, and reviewed the manuscript. AN and GA designed the experiment, oversaw the sample collection, and reviewed the manuscript. YW and PS reviewed the qPCR data. TK designed the experiment, reviewed and edited the manuscript. RG designed the experiment, reviewed the manuscript, and was also part of the project veterinary personnel. HZ designed the experiment, oversaw the data analyses and sample collection, and reviewed and edited the manuscript. SL contributed to the experimental design, edited and reviewed manuscript. JD designed the experiment, oversaw the data analyses and sample collection, and edited and reviewed manuscript. All the authors contributed to the article and approved the submitted version.

## Conflict of Interest

The authors declare that the research was conducted in the absence of any commercial or financial relationships that could be construed as a potential conflict of interest.

## Footnotes

1 https://www.ncbi.nlm.nih.gov/genome/gdv/

2 http://en.climate-data.org/
